# Investigation of the Influence of Structure, Stoichiometry, and Synthesis Temperature on the Optical Properties of CdTe Nanoplatelets

**DOI:** 10.3390/nano14221814

**Published:** 2024-11-13

**Authors:** Aigerim Ospanova, Yerkebulan Koshkinbayev, Asset Kainarbay, Temirulan Alibay, Rakhima Daurenbekova, Aizhan Akhmetova, Alexander Vinokurov, Sergei Bubenov, Sergey Dorofeev, Dulat Daurenbekov

**Affiliations:** 1 Institute of Physical and Technical Sciences, L.N. Gumilyov Eurasian National University, Kazhymukan Str., 13, 010000 Astana, Kazakhstan; aygerim-ospanova-00@mail.ru (A.O.);; 2 Department of Chemistry, Lomonosov Moscow State University, Leninskie Gory, 1–3, 119991 Moscow, Russia

**Keywords:** nanoplatelets, stoichiometry, TXRF, colloidal synthesis, electron diffraction

## Abstract

Colloidal cadmium telluride (CdTe) nanoplatelets (NPLs) are promising materials for optoelectronic applications, such as photovoltaics and light-emitting diodes, due to their unique optical and electronic properties. However, controlling their growth, thickness, and stoichiometry remains challenging. This study explores the effect of synthesis temperature on the structural, optical, and stoichiometric properties of CdTe NPLs. CdTe NPLs were synthesized at temperatures of 170 °C, 180 °C, 190 °C, and 200 °C using colloidal methods. The resulting NPLs were characterized by UV–Vis absorption spectroscopy, photoluminescence (PL) spectroscopy, transmission electron microscopy (TEM), and total reflection X-ray fluorescence (TXRF) to assess their morphology, structure, and elemental composition. The results showed that the synthesis temperature significantly affected the NPL’s morphology and stoichiometry. Optimal stoichiometry was achieved at 180 °C and 190 °C, with the crystal structure transitioning from zinc blende at lower temperatures to wurtzite at higher temperatures. Optical properties, including luminescence intensity and emission peaks, also varied with temperature. The synthesis temperature is an important parameter in controlling the structural and optical properties of CdTe NPLs. The optimal conditions for obtaining NPLs with the best characteristics were identified at 190 °C, presenting important findings for further optimization of CdTe NPL synthesis for optoelectronic applications.

## 1. Introduction

Among all quasi-two-dimensional cadmium chalcogenides, nanobelts, and nanoshells, which are known as nanoplatelets (NPLs), have a controlled uniform thickness. Due to this thickness, they have narrow exciton bands and transitions of 5–8 nm in width [1]. By creating core-shell heterostructures and solid-solution nanoparticles, it is possible to control and study their optical properties [2].

Colloidal semiconductor NPLs have a thin thickness along which exciton movement is limited, resulting in a narrow photoluminescence band and large nonlinear absorption cross-section [3], and are a promising option for creating light-emitting diodes with ultra-high color purity through solution processing [4]. Today, the structure of colloidal quantum dots CdSe of wurtzite crystal is best known and has been investigated since the photoluminescence can be controlled in the visible wavelength region. Alternatives, such as zinc blende, CdTe, CdS, ZnSe, and ZnS, are being investigated to extend the range [5,6,7].

Cadmium telluride, a direct bandgap semiconductor of group A^II^B^VI^ with a zinc blende structure, is used as an active material in thin-film solar cells, which have attracted interest in photovoltaics due to the continuous advancement in their performance [8]. Nanowire structures offer significant advantages in terms of their anti-reflective and light-trapping effects, as well as reduced recombination losses due to shortened transport paths for photogenerated carriers [9]. CdTe colloidal nanoplatelets demonstrate a high absorption coefficient value; however, their emission efficiency is low. Nevertheless, these NPLs can be employed in radiation sensing applications due to their heavy metal-based composition [10]. However, controlling the thickness growth of telluride- and selenide-based NPLs remains an important challenge. In NPLs, the lifetime of exciton states determines the optical gain threshold [11,12]. CdTe, due to the creation and formation of free excitons, has a large absorption cross-section [13,14]; the multi-exciton lifetime of cadmium chalcogenide NPLs (hundreds of ps) is longer than that of QDs (10s of ps) with similar exciton transition energy [15,16].

The authors of [17] found that for CdTe, CdSe, and CdS nanocrystals, the size affects the extinction coefficient per mole of nanocrystals in the exciton absorption peak. In determining the size-dependent properties of semiconductor nanocrystals, quantum confinement is important, which is associated with the main effect of the size-dependent extinction coefficient of nanocrystals, which has been studied so far for CdSe NPLs [18], but much less studied for CdTe NPLs.

In this paper, we investigated a robust synthesis method to study the growth and optical properties of CdTe NPLs. The authors of [19] demonstrated the colloidal synthesis of CdTe semiconductor nanoplatelets. They obtained a series of nanoplatelets with different thicknesses, exhibiting the first exciton peak at wavelengths of 428 nm, 500 nm, and 556 nm. They tried to control the shape and size of CdTe NPLs and investigated the dependence on cadmium precursors and ligands. They also focused on the introduction of the tellurium precursor and studied the influence of the temperature at which it was injected. Recently, the synthetic route of CdSe NPLs has been extended to CdTe systems [20]. In many studies, the optical properties have been investigated, but the stoichiometry of the samples has not been considered. In article [21], the surface chemistry and optoelectronic properties of CdSe nanoplatelets were researched, and the ratio of cadmium-to-selenium was determined based on Rutherford Backscattering Spectrometry (RBS) data. However, for CdTe nanoplatelets, such dependence has not been studied.

In this article, we demonstrate the synthesis of colloidal CdTe nanoplatelets, analyzing them using optical spectroscopy, transmission electron microscopy, and TXRF methods. Additionally, the dependence between synthesis temperature, NPL structure, and stoichiometry is examined.

## 2. Materials and Methods

### 2.1. Chemicals

The chemicals used were 1-Octadecene (ODE) (Sigma-Aldrich, St. Louis, MO, USA, 90%), propionic acid (Sigma-Aldrich, 99%), oleic acid (OA) (Sigma-Aldrich 90%), cadmium acetate dihydrate (Cd(OAc)_2__2(H_2_O)), (>98%), tellurium powder (Te) (98%), trioctylphosphine (TOP) (95%), hexane (95%), ethanol (95%), and acetone (90%).

### 2.2. Precursor Preparation

Cadmium propionate Cd(Prop)_2_: To prepare the cadmium propionate precursor, 8.07 mmol of cadmium acetate and 10 mL (133 mmol) of propionic acid were added in a 50 mL three-neck flask, stirred at 90 °C, and degassed under argon for one hour. After one hour, the mixture was removed from heat and cooled. At room temperature, 20 mL of acetone was added until the solution turned turbid, and the precipitate was filtered and dried. White cadmium propionate powder C_6_H_10_CdO_4_ was obtained.

### 2.3. Synthesis of NPL CdTe

In a 50 mL three-neck flask, 0.5 mmol of cadmium propionate, 0.25 mmol of oleic acid, and 10 mL of ODE were added, heated to 95 °C, stirred, and degassed under argon for 2 h. After 2 h, the temperature was raised to 170 °C, and at the synthesis temperature, 100 μL of 1 M TOP-Te in TOP solution, pre-diluted with 0.5 mL of ODE, was injected. Upon injection, the color changes to dark yellow and then to orange. Afterward, the mixture is removed from heat and cooled in cold water, and the precipitate is collected by centrifuging at 5000 rpm for 5 min. This synthesis process was performed several times, increasing the temperature by 10 degrees each time: 170 °C, 180 °C, 190 °C, and 200 °C. With each following synthesis, the color became progressively darker.

For the synthesis of CdTe nanoplatelets, which is typical for colloidal methods, the following key reactions are commonly used:

1. The reaction between cadmium acetate dihydrate and propionic acid:
  $$Cd(CH_{3}COO{)}_{2}\cdot 2H_{2}O+2C_{2}H_{5}COOH\to Cd(C_{2}H_{5}COO{)}_{2}+2CH_{3}COOH↑+2H_{2}O$$
2. $Cd(C_{2}H_{5}COO{)}_{2}+Te-TOP\to CdTe(NPLs)$

The synthesis is carried out in an ODE solvent under an inert atmosphere.

### 2.4. Material Characterization

The UV–Vis absorption spectrum of the NPLs was recorded using a Jasco V-770 spectrophotometer (JASCO Corporation, Tokyo, Japan) in the range of 200–800 nm at room temperature. Photoluminescence (PL) measurements at room temperature for samples synthesized at different temperatures were performed in quartz cuvettes using a Solar CM 2203 spectrofluorimeter (SOLAR Ltd., Minsk, Belarus). Chemical analysis was carried out, and the elemental composition of CdTe NPL was studied using the TXRF method on the S2 Picofox device (Bruker Nano GmbH, Berlin, Germany). For this, the concentration of Cd in the composition of the NPLs in hexane sols was preliminarily estimated using UV–Vis absorption spectroscopy Jasco V-770. Then, 3 to 15 μL of the sol (containing approximately 100 ng of cadmium) was applied to a sapphire substrate using a micropipette and dried. As a result of the analysis, the absolute masses of the elements in the sample were determined, and the concentrations of cadmium and tellurium in the original sols were refined. Electron microscopy and electron diffraction studies were conducted using a JEM-2100 (JEOL) TEM (JEOL Ltd., Tokyo, Japan) equipped with a LaB_6_ gun and an 11-megapixel Quemesa CCD camera (Olympus, Tokyo). The accelerating voltage was set to 200 kV. Hexane sols containing NPLs were deposited onto carbon-coated copper grids and dried. The grids were then etched in argon plasma to remove organic material before examination. The sizes of the NPLs were manually measured from micrographs using Image-Pro Plus 6.0 software.

## 3. Results and Discussion

We studied the dependence of NPL growth on the synthesis temperature, as well as the stoichiometric yield of Cd and Te in the nanoplatelets. The nucleation process at different synthesis temperatures was studied. As the starting materials for the experiments, 3.5 ML CdTe NPL samples, previously synthesized according to the established methodology [19], were used. We investigated the effect of temperature on the lateral expansion of NPLs in the range of 170–200 °C while the other synthesis parameters remained constant.

The morphology, structure, and stoichiometry of CdTe NPL samples synthesized at different temperatures were studied. The analysis of the NPL samples obtained using XRF shows varying stoichiometry (Cd-to-Te) at different temperatures: 2.16 at 170 °C, 1.343 at 180 °C, 1.374 at 190 °C, and 0.865 at 200 °C, which corresponds with the data reported in the literature [3,10,22]. The best stoichiometry was observed in the samples at 180 °C and 190 °C, with a ratio of approximately ~1.33 (Table S1), which is determined by the concentrations of Cd and Te. In the sample synthesized at 200 °C, an excess of tellurium compared to cadmium can be observed [21].

**Figure 1:**
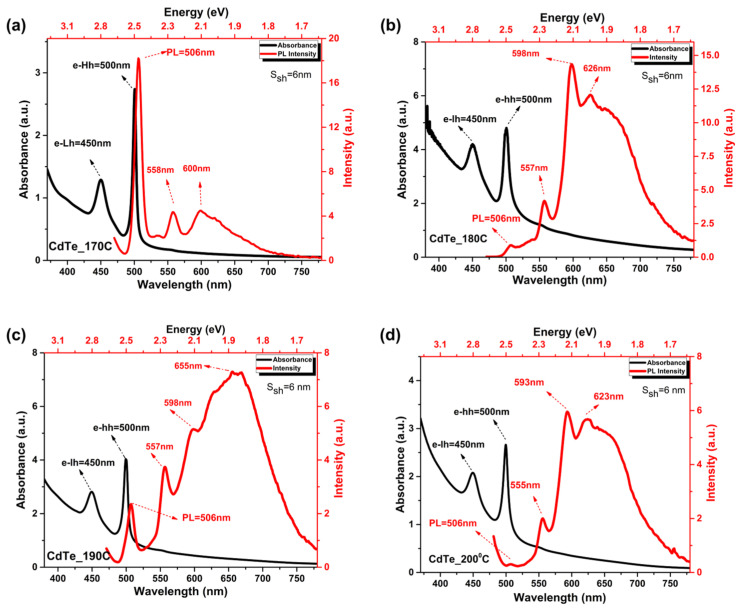
Absorption and luminescence spectra of CdTe at temperatures of (**a**) 170 °C, (**b**) 180 °C, (**c**) 190 °C, and (**d**) 200 °C.

The formation of CdTe NPLs at various temperatures was studied using optical absorption and photoluminescence spectra, as shown in Figure 1.

All graphs showed two main peaks at around 450 nm and 500 nm, corresponding to electron transitions to the light-hole (e-Lh) and heavy-hole (e-Hh) levels, respectively. These peaks did not shift across different synthesis temperatures. The full-width at half maximum (FWHM) also remained consistent with variations in synthesis temperature. For excitonic absorption related to heavy holes, the FWHM was 6.3 nm, and for light holes, it was 25.6 nm.

For the synthetic temperature of 170 °C, excitonic luminescence is observed at 506 nm, with an excitation wavelength of 450 nm, along with defect-related bands with maxima at 558 nm and 600 nm (Figure 1a). For the 180 °C sample, quenching of the excitonic band at 506 nm occurs with a sharp increase in intensity at 600 nm (Figure 1b). Rearrangement of the bands can be observed in the case of 190 °C, with the dominance of a broad long-wavelength band, while at 200 °C, complete quenching of the excitonic band occurs (Figure 1c,d). The luminescence peaks associated with the defect band in NPLs are somewhat understudied and remain an interesting subject for further research [23].

**Figure 2:**
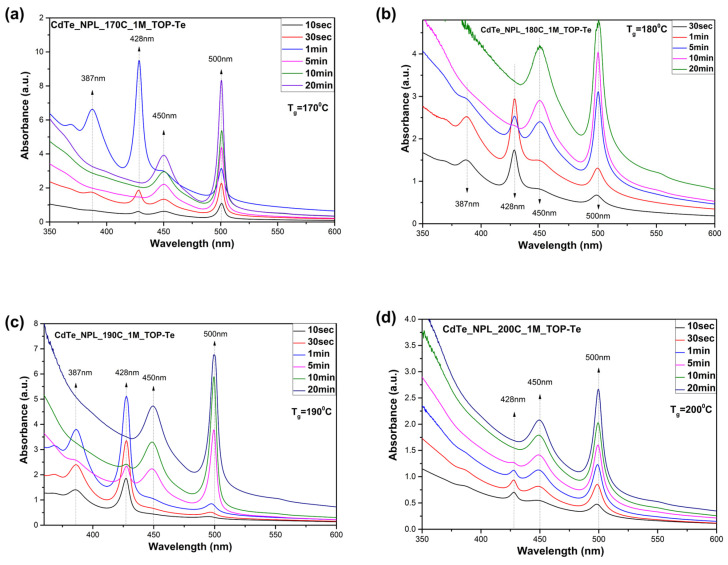
Absorption spectra of the main CdTe samples and aliquots taken at different minutes during the reaction. (**a**) 170 °C; (**b**) 180 °C; (**c**) 190 °C; (**d**) 200 °C.

A broad emission peak was previously observed for CdTe nanoplatelets [6,19] and other nanocrystals [24,25] commonly linked to trap states emission. For HgTe nanoplatelets, the formation of charge carriers was previously identified, leading to the appearance of a sharp emission peak that is readily ascribed to a 4-monolayer structure [26]. Here, a similar hypothesis is refuted due to the sharpness of the peak and the stability of the emission over time at elevated temperatures. Consequently, the peak at 558 nm is associated with 4 monolayers, which form simultaneously with the growth of 3 monolayers. It is noteworthy that although the excitonic feature of the 4-monolayer ensemble is barely evident in the absorption spectrum, it strongly influences the emission spectrum [23]. Figure 2 shows the absorption spectra of aliquots taken at different time intervals upon the addition of TOP-Te for NPL growth.

Within the first 30 s after precursor addition, the formation of NPLs is not observed, after which two absorption peaks appear with maxima at 450 nm and 500 nm [20]. The nucleation of nanoparticles is achieved at the initial stage of injection, leading to the formation of thin NPLs [27].

The optical absorption spectrum demonstrates a shift in absorption peaks with the increasing thickness of the NPLs. If the sample contains multiple populations of NPLs with different thicknesses, the resulting spectrum resembles a linear combination of discrete elements [28]. At the initial stage of the synthesis, an additional absorption peak appears at 387 and 428 nm (Figure 2), which is consistently observed across all samples and corresponds to the early stage of nucleation. At the initial phase of injection, supersaturation of the concentration occurs, stimulating nucleation, which leads to the initial formation of thin nanoplatelets [19], which subsequently dissolve and serve as a building material for the growth of thicker NPLs of 3.5 ML (Figure 2a–c). According to TEM images, other NPL populations are not observed in the samples. Thus, the concentration at low temperatures is excessive, whereas, at higher temperatures, it becomes suitable for the growth of NPLs. These assumptions align with the findings of the author of [27], who indicated that, according to TEM data, new populations or smaller-sized nanoparticles were not detected during the early stages of synthesis. Five minutes after injection, as the reaction time increases, the initial peaks begin to disappear, and the two main peaks (450 and 500 nm), corresponding to the growth of the nanoplatelets, become more clear. It suggests that the thin nanoplatelets begin to aggregate into larger, thicker nanoplatelets.

**Figure 3:**
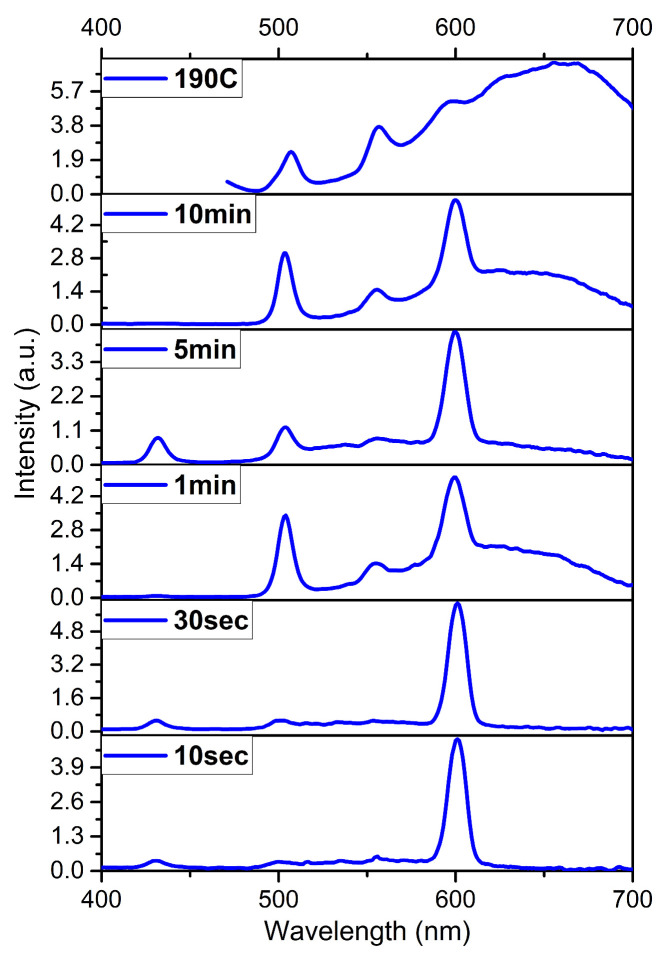
The luminescence spectra of aliquots measured at different times during the reaction, and the final reaction mass synthesized at 190 °C.

A sample with the best stoichiometry was identified at 190 °C. The change in the luminescence spectrum at different synthesis times at 190 °C is shown in Figure 3.

Figure 3 represents luminescence spectra measured at different time intervals and a temperature of 190 °C. The main luminescence peak is observed around 500 nm, and this peak is associated with the excitonic luminescence of CdTe nanoplatelets, occurring due to transitions to the ground state. The narrow peak at 600 nm, which appears 10 s after injection, can be interpreted as the result of the formation of initial CdTe nanocrystals, as investigated in the work of the author [29]. This peak indicates transitions between energy levels associated with a specific initial thickness or configuration of the nanoplatelets. Over time, this peak broadens, which may be linked to changes in the nanoplatelet size and/or the growth of nanostructures, as well as the development of various surface defects. The peak at 558 nm, appearing after 1 min of synthesis, probably indicates the formation of thinner layers or the appearance of an additional phase modification in the CdTe nanoplatelets. In the work of the author of [23], a sharp peak at 550 nm and a broad peak around 615 nm also appeared in the photoluminescence spectrum, in addition to the peak at 500 nm, and these were attributed to emission trap states.

**Figure 4:**
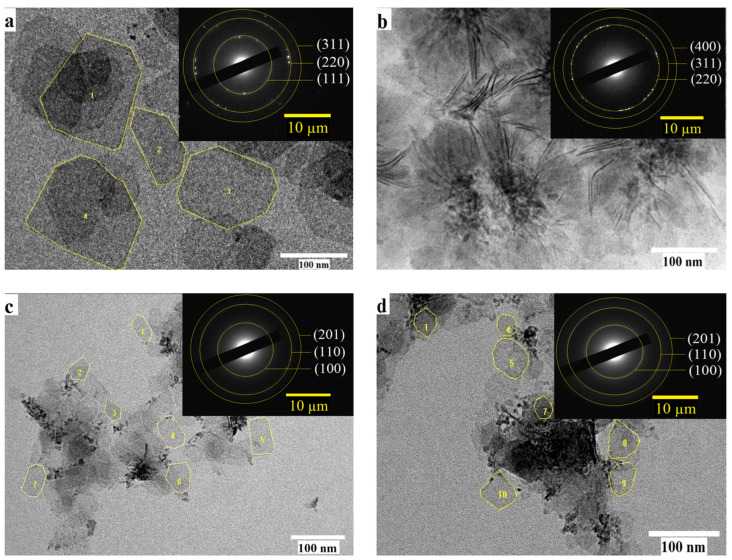
TEM images of NPLs. (**a**) 170 °C; (**b**) 180 °C; (**c**) 190 °C; (**d**) 200 °C.

To investigate the structural characteristics of the NPLs and to understand their growth mechanism, purified NPLs were studied. The morphology and selected area electron diffraction (SAED) were studied using high-resolution TEM (HRTEM), the results of which are shown in Figure 4.

Distinct rectangular NPLs are observed, retaining their two-dimensional shape with minimal edge degradation (Figure 4). Selected area electron diffraction (SAED) for CdTe NPLs demonstrated a cubic zinc blende structure (Figure 4, inset in a, b). (111), (220) and (311) at temperature 170 °C and (220), (311), (400) at 180 °C [30]. Table 1 shows the data of SAED with interplanar distances and Miller indices for NPL CdTe obtained using TEM.

In the work in [28], the growth of nanoplatelets with various cadmium precursors was investigated, and their optical characteristics, along with the influence of temperature and ligands on the lateral growth of NPLs, were examined. The cubic structure of CdTe nanoplatelets synthesized at a temperature of 180 °C was also established. However, the structure of such nanoplatelets at temperatures above 180° C has not been studied. After conducting the synthesis at 170–200 °C, we found that at low temperatures such as 170 °C and 180 °C the structure remains cubic. This corresponds to the literature data; however, at higher temperatures, such as 190 °C and 200 °C, the structure changed to wurtzite, the measurements of which are shown in Table 1 (SAED in the inset of Figure 4c,d). It is assumed that a higher temperature leads to the formation of the wurtzite structure. In the work of the authors of [30], the wurtzite structure of CdTe NPL was also investigated. The small size of the obtained particles and the appearance of various microstructures in the solution at 180 °C (Figure 4b) suggest significant surface stabilization by long-chain surfactants and a high degree of supersaturation, which is maintained throughout the extended synthesis duration.

**Figure 5:**
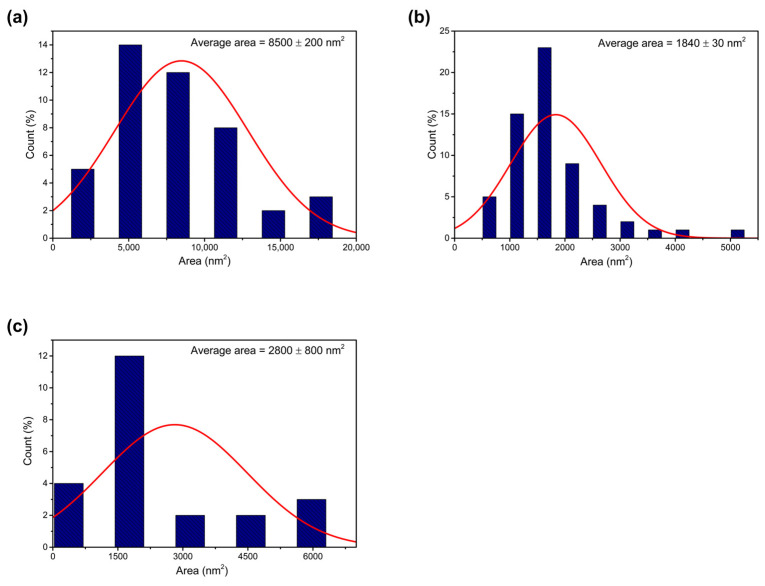
Histograms of the area of CdTe nanoplatelets: samples obtained at (**a**) 170 °C, (**b**) 190 °C, and (**c**) 200 °C.

The dimensions of the NPLs from HRTEM images for the samples synthesized at 170 °C, 190 °C, and 200 °C were calculated using the Image-Pro Plus 6.0 software and are shown in Figure 5.

Figure 5 shows the histograms of the dimensions of CdTe nanoplatelets. For an initial temperature of 170 °C, the average area of the NPL is 8500 ± 200 nm^2^; for 190 °C, the average area is 1840 ± 30 nm^2^; and for 200 °C, the average area is 2800 ± 800 nm^2^. The histogram for 180 °C is not obtained, as it is not possible to identify the NPL from the TEM images, and consequently, their average area cannot be determined.

A comparative analysis of the characteristic data is presented in Table 2.

As shown in Table 2, the samples synthesized at 180 °C and 190 °C are the most optimal in terms of stoichiometry. However, based on the TEM images, the 180 °C sample is not appropriate. Rectangular nanoplatelets with well-defined edges were obtained at 170 °C, 190 °C, and 200 °C, which also differ in structure. Thus, based on the data in Table 1 and Table 2, we concluded that the optimal temperature for obtaining 3.5 ML nanoplatelets with a wurtzite structure is 190 °C.

## 4. Conclusions

This study highlights the significant impact of synthesis temperature on the structural, stoichiometric, and optical properties of CdTe nanoplatelets, identifying 190 °C as the optimal temperature for producing high-quality 3.5 ML NPLs with high homogeneity and precise stoichiometry of ~1.33. Rectangular nanoplatelets with sharp edges were obtained, and an increase in temperature led to a shift in their crystalline phase from cubic to wurtzite. An in-depth analysis of stoichiometry’s effect on optical properties, rarely addressed in similar studies, demonstrates the value of TXRF analysis for precise compositional control. The obtained data create opportunities for improving synthesis methods, promoting the development of efficient nanomaterials for applications in optoelectronics and photonics, and prospects for the development of next-generation LEDs and optical devices with high color purity and operational stability.

## Figures and Tables

**Table 1:** Data obtained using SAED.

CdTe NPL	d-Spacing (Å)	Miller Indices (hkl)	d (Bulk) (Å)
170 °C	3.8	(111)	3.74
	2.3	(220)	2.29
	1.9	(311)	1.95
180 °C	2.3	(220)	2.29
	2.0	(311)	1.95
	1.7	(400)	1.619
190 °C	4.0	(100)	3.98
	2.3	(110)	2.29
	1.9	(201)	1.919
200 °C	4.0	(100)	3.98
	2.3	(110)	2.29
	1.9	(201)	1.919

**Table 2:** Comparison of all samples according to stoichiometry data, TEM images, and structure.

Temperature, (°C)	Stoichiometry	TEM Images (Morphology)	Average Area of NPL (nm^2^)	Structure
170 °C	2.16	NPL	8500 ± 200	Sphalerite
180 °C	1.343	Coagulate	-	Sphalerite
190 °C	1.374	NPL	1840 ± 30	Wurtzite
200 °C	0.865	NPL	2800 ± 800	Wurtzite

## Data Availability

The original contributions presented in the study are included in the article/supplementary material; further inquiries can be directed to the corresponding author/s.

## Supplementary Materials

The following supporting information can be downloaded at https://www.mdpi.com/article/10.3390/nano14221814/s1, Table S1: Stoichiometric data of CdTe NPL obtained using the TXRF method.
