# Supplementary material for: Investigation of the Influence of Structure, Stoichiometry, and Synthesis Temperature on the Optical Properties of CdTe Nanoplatelets
Source: Nanomaterials (Basel). 2024 Nov 13;14(22):1814. doi: 10.3390/nano14221814 (PMC11597514; doi:10.3390/nano14221814)
Supplement: Supplementary file 1 [file nanomaterials-14-01814-s001.zip › Supporting information.pdf]

Supporting information

Investigation of the Influence of Structure, Stoichiometry, and Synthesis Temperature on the Optical Properties of CdTe Nanoplatelets

Aigerim Ospanova [1], Yerkebulan Koshkinbayev [1], Asset Kainarbay [1], Temirulan Alibay [1], Rakhima Daurenbekova[1], Aizhan Akhmetova [1,*], Alexander Vinokurov [2], Sergei Bubenov [2], Sergey Dorofeev [2] and Dulat Daurenbekov [1,*]

[1] Institute of Physical and Technical Sciences, L.N. Gumilyov Eurasian National University, Kazhymukan Str., 13, Astana, Kazakhstan; aygerim-ospanova-00@mail.ru (A.O)
[2] Department of Chemistry, Lomonosov Moscow State University, Leninskie Gory, 1–3, 119991 Moscow, Russia, bubenovss@my.msu.ru (S.B.)
* Correspondence: akhmetova_as_3@enu.kz (A.A.); 900902399020@enu.kz (D.D.)

Table S1 Stoichiometric data of CdTe NPL obtained using TXRF method

	m_{Cd} (ng)	m_{Te} (ng)	ν_{Cd} (nmol)	ν_{Te} (nmol)	C(Cd)/ C(Te)
CdTe 170^{0}C	131,51	69,19	1,17	0,542	2,16
CdTe 180^{0}C	139,28	117,69	1,239	0,922	1,343
CdTe 190^{0}C	136,83	113,13	1,217	0,886	1,374
CdTe 200^{0}C	150,79	197,79	1,341	1,55	0,865
